# Inhibition of pathologic immunoglobulin E in food allergy by EBF-2 and active compound berberine associated with immunometabolism regulation

**DOI:** 10.3389/fimmu.2023.1081121

**Published:** 2023-02-07

**Authors:** Nan Yang, Anish R. Maskey, Kamal Srivastava, Monica Kim, Zixi Wang, Ibrahim Musa, Yanmei Shi, Yixuan Gong, Ozkan Fidan, Julie Wang, David Dunkin, Danna Chung, Jixun Zhan, Mingsan Miao, Hugh A. Sampson, Xiu-Min Li

**Affiliations:** ^1^ General Nutraceutical Technology, Elmsford, NY, United States; ^2^ Department of Pathology, Microbiology and Immunology, New York Medical College, Valhalla, NY, United States; ^3^ Department of Pediatrics, Icahn School of Medicine at Mount Sinai, New York, NY, United States; ^4^ Department of Allergy, Peking Union Medical College Hospital, Beijing, China; ^5^ Academy of Chinese Medicine Sciences, Henan University of Traditional Chinese Medicine, Zhengzhou, Henan, China; ^6^ Division of Hematology and Medical Oncology, Icahn School of Medicine at Mount Sinai, New York, NY, United States; ^7^ Department of Biological Engineering, Utah State University, Logan, UT, United States; ^8^ Department of Bioengineering, Abdullah Gul University, Kayseri, Türkiye; ^9^ Department of Medicine, Icahn School of Medicine at Mount Sinai, New York, NY, United States; ^10^ Department of Otolaryngology, New York Medical College, Valhalla, NY, United States

**Keywords:** berberine, IgE, food allergy, metabolism, anaphylactic allergic reaction

## Abstract

**Introduction:**

Food allergy is a significant public health problem with limited treatment options. As Food Allergy Herbal Formula 2 (FAHF-2) showed potential as a food allergy treatment, we further developed a purified version named EBF-2 and identified active compounds. We investigated the mechanisms of EBF-2 on IgE-mediated peanut (PN) allergy and its active compound, berberine, on IgE production.

**Methods:**

IgE plasma cell line U266 cells were cultured with EBF-2 and FAHF-2, and their effects on IgE production were compared. EBF-2 was evaluated in a murine PN allergy model for its effect on PN-specific IgE production, number of IgE^+^ plasma cells, and PN anaphylaxis. Effects of berberine on IgE production, the expression of transcription factors, and mitochondrial glucose metabolism in U266 cells were evaluated.

**Results:**

EBF-2 dose-dependently suppressed IgE production and was over 16 times more potent than FAHF-2 in IgE suppression in U266 cells. EBF-2 significantly suppressed PN-specific IgE production (70%, p<0.001) and the number of IgE-producing plasma cells in PN allergic mice, accompanied by 100% inhibition of PN-induced anaphylaxis and plasma histamine release (p<0.001) without affecting IgG1 or IgG2a production. Berberine markedly suppressed IgE production, which was associated with suppression of XBP1, BLIMP1, and STAT6 transcription factors and a reduced rate of mitochondrial oxidation in an IgE-producing plasma cell line.

**Conclusions:**

EBF-2 and its active compound berberine are potent IgE suppressors, associated with cellular regulation of immunometabolism on IgE plasma cells, and may be a potential therapy for IgE-mediated food allergy and other allergic disorders.

## Introduction

Food allergy (FA) has rapidly increased over the past 2 decades affecting 32 million Americans, with annual costs of $25 billion (1–9). FA anaphylaxis, a potentially life-threatening condition, increased 200-400% in toddlers to teens (10) accounting for up to 81% of pediatric anaphylaxis (1). Peanut allergies are lifelong and cause severe reactions and there is currently no cure (1, 10–15). Common treatments, such as prophylactic-food avoidance, or therapeutic-food allergen oral immunotherapy (OIT) are limited and impractical (16–22). Therefore, there is a significant need for safe, effective, and non-food restricted therapeutics. FA is primarily mediated by food protein specific immunoglobulin E (sIgE) (23). IgE-producing long-lived plasma cells (IgE^+^LPC) cause “lifelong allergy” (24–26). Persistent IgE is a significant barrier to FA mitigation. Omalizumab, an anti-IgE antibody, “traps” IgE but does not target its production. OIT, including Palforzia^®^, does not decrease IgE production, but in fact may paradoxically increase IgE levels, carrying a significant immune reaction risk (27–32). Therefore, a safe and effective therapy that targets excess IgE production represents an important strategy for food allergy treatment.

In recent years, a substantial number of findings have been made in the area of immunometabolism, the changes in intracellular metabolic pathways in immune cells that alter their function (33). Glycolysis is one of the major metabolic pathways involved in immune cell regulation. Immunoglobulins are glycoproteins that are produced, glycosylated, and secreted in the endoplasmic reticulum (ER), requiring energy and metabolites. Mitochondria are a highly efficient organelle for fueling the ER through ATP consumption *via* oxidation (respiration) (34). Alternative energy (ATP) production is *via* glycolysis though pyruvate. However, this pathway generates less ATP than mitochondrial oxidation (3 vs. 31 ATP molecules). IgE is the most heavily glycosylated isotype, with sugar moieties accounting for 12-14% of its molecular weight compared to approximately 3% for IgG (35–38). As more than 90% of glucose uptake in plasma cells (PCs) is utilized for antibody glycosylation, IgE^+^PCs face greater metabolic stress compared to other isotype PCs. It was reported that inhibition of glycosylation essentially shuts down IgE release while IgG is largely unaffected (39, 40). Plasma cells require high energy and are regulated by specific transcription factors. XBP1 expression, which is high in plasma cells, is important for plasma cell differentiation (41) and secretory function (42, 43). XBP1 promotes gene expression involved in mitochondrial and ER biogenesis (44) and is required for antibody generation of heavy and light chain (IgH/IgL) transcripts (45). Loss of XBP-1 is associated with complete absence of plasma cells and circulating immunoglobulins (46, 47). This is due to inefficient processing and exportation of immunoglobulins and accumulation of unfolded proteins further contributing to ER stress (48).Thus, modulating XBP1 and mitochondrial glucose metabolism may affect IgE^+^ PC antibody production and secretion (44), but direct evidence is limited.

Like FA, immunologic responses in parasite infections are associated with excessive production of IgE. FAHF-2 derived from *Fructus mume formula*, which has been used to treat parasite infection traditionally, showed a reduction of peanut specific IgE and protected against anaphylaxis in murine models of peanut allergy (49–53), suggesting a possible FA treatment. A new purification method was developed using ethyl acetate and butanol to concentrate active ingredients. The objective of this study is to investigate the potency of EBF-2 on IgE production *in vitro* and *in vivo*, and to understand underlying mechanisms on immunometabolism regulation of IgE producing cells. We first compared the effects of EBF-2 and FAHF-2 on IgE production across-multiple batches using IgE producing plasma cells and chromatographic approaches. We also assessed EBF-2’s effects on PN-specific IgE production, IgE^+^PC count, and anaphylactic symptoms in a murine peanut allergy model. Furthermore, we investigated the effect of the bioactive compound berberine (BBR) on XBP1 expression and mitochondrial metabolism in a human IgE^+^PC line.

## Material and methods

### EBF-2 constituents, and production

EBF-2 was generated by purification of FAHF-2 with a safe solvents consisting of butanol and ethyl acetate (52). 8 herb constituents (*Prunus mume*, *Zanthoxylum schinifolium*, *Angelica sinensis*, *Zingiber officinalis*, *Cinnamomum cassia*, *Phellodendron chinense*, *Coptis chinensis*, and *Panax ginseng*) were extracted using butanol, while the *Ganoderma lucidum* was extracted using ethyl acetate and the dried extracts were combined to generate the EBF-2 powder substance. Three batches of FAHF-2 and EBF-2 were tested in this study-manufacturing date and shelf life are listed in **Supplemental Table 1**. Botanical information for individual herbs, including geographical location, harvest season, pre-processing, heavy metal and pesticide residues, and quality control methods, have been published previously (54).

### Cell culture and IgE measurement

The IgE-producing human plasma cell line U266 (ATCC, MD) was grown in complete media containing RPMI 1640 medium supplemented with 10% FBS, 1 mM sodium pyruvate, 1×10^-5^ M β-ME and 0.5% penicillin-streptomycin (55, 56) at 2×10^4^ cells/mL in 48 well plates. Three batches of FAHF-2 and 3 batches of EBF-2 at serial dilution concentrations starting at 500 μg/mL and 120 μg/mL, respectively, were used in culture for 6 days. Berberine (purity>98%, Sigma Aldrich, St Louis, MO) at serial concentrations starting at 5 μg/mL were also tested on U266 cells. Supernatants were harvested, IgE levels were determined by ELISA (Mabtech Inc, OH) and cell viability was evaluated by trypan blue exclusion (55).

### Mice, peanut sensitization, and EFB-2 treatment

**Figure 1:**
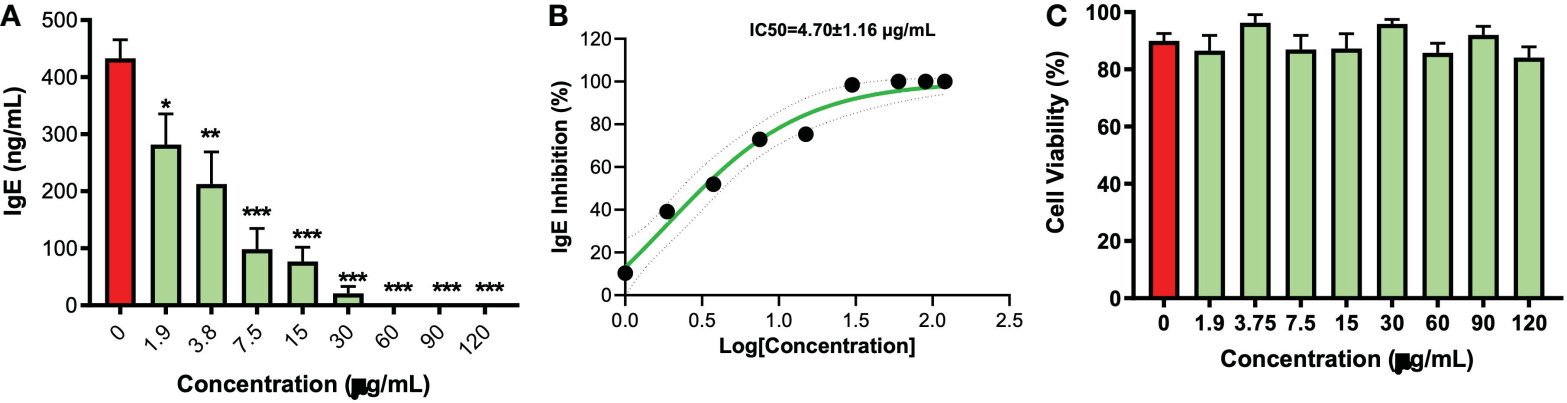
Inhibitory effect of EBF-2 on IgE production by plasma cell line U266 cells. **(A)** U266 cells were treated with EBF-2 at different concentrations and cultured for 6 days. Supernatants were collected, and IgE levels were determined by ELISA. **(B)** IC_50_ values for EBF-2 were calculated to be IC50=4.70±1.16 μg/mL. IC_50 =_ 4.70 ± 1.16 mg/mL **(C)** Cell viability was measured by trypan blue excursion showed no cell cytotoxicity. IgE levels are expressed as Mean± SEM, and significance is indicated by *p≤0.05, **p≤0.01, and ***p≤0.001 as compared to the untreated control. N=9 independent cultures over 3 batches.

Six-week-old female C3H/HeJ mice purchased from the Jackson Laboratory (Bar Harbor, ME) were maintained in pathogen-free facilities at the Mount Sinai vivarium according to standard guidelines (57). Mice were intragastrically (i.g.) sensitized with 10 mg of homogenized peanut (PN) in 0.5 mL PBS containing 75 mg sodium bicarbonate, 10 µg of the mucosal adjuvant cholera toxin (CT) (List Laboratories, Campbell, CA), and 16.5 µL (1.1 µL/g body weight) of 80 proof Stolichnaya Vodka^®^ (a source of food grade ethanol) to neutralize stomach pH and increase gastrointestinal permeability, three times during week 0 (50). Thereafter, sensitization was done weekly as above except that the CT dose given was 20 µg. The boosting dose of 50 mg PN was given at weeks 6 and 8 using the same gavage solution as in weeks 1 through 5. These mice were defined as peanut allergic (PNA) mice. One day following the last boost, at which hypersensitivity was developed (58), PNA mice received EBF-2 treatment at 3.84 mg in 0.5 mL drinking water, twice a day for 4 weeks. One week after completing the treatment, mice were challenged with ground peanut (200 mg) i.g. and again 4 weeks later. Anaphylactic reactions were accessed (**Figure 1A**). Sham treated PNA mice and naïve mice (unsensitized/untreated) were used as disease and normal controls, respectively. In a separate experiment, to determine the persistent impact of EBF-2 on IgE producing cells by flowcytometry analysis, EBF-2 treated, and sham treated PNA mice received periodic oral exposure of either boiled (10 mg/mouse) or roasted peanut (200 mg/mouse) approximately every 10-15 weeks. Mice were terminated using ketamine/xylazine euthanasia protocol. Briefly, mice were given over-dose (15µL/g body weight) of ketamine-xylazine mixture (100mg/mL and 10 mg/mL respectively) intraperitoneally. After the mice were in deep anesthetics, blood samples were collected, and mice were sacrificed by cervical dislocation after which tissue samples were collected. All animal experiments were approved and performed according to the instruction and guidelines of the Institutional Animal Care and Use Committee (IACUC) of Icahn School of Medicine at Mount Sinai.”

### Assessment of hypersensitivity reactions

Symptoms were evaluated 30-40 minutes following oral PN challenge as described previously (51, 59), and symptoms were scored utilizing the scoring system ranging from 0 (no reaction) to 5 (fatal reactions), described previously (59). Rectal temperatures were also measured immediately after scoring using a rectal probe (Harvard Apparatus, Holliston, MA).

### Measurement of plasma histamine levels

Blood samples were collected *via* sub-mandibular bleeding 30 minutes after scoring and measurement of body temperature following oral peanut challenge. Plasma was harvested within 20 minutes after blood collection and stored at -80°C until analyzed. Histamine was measured using a commercial enzyme immunoassay kit (Fisher Scientific, NJ) as described by the manufacturer (58).

### Measurement of serum peanut specific-IgE, IgG1 and IgG2a

PN-specific-IgE, IgG1 and IgG2a in serum was measured as reported previously (50, 51, 59). Briefly, microtiter plates were coated with peanut extract (sample wells), anti-mouse IgE (BD Biosciences, San Jose, CA, for IgE reference wells), or DNP-HSA (Sigma-Aldrich for IgG2a and IgG1 reference wells) and incubated overnight at 4° C. Subsequently, the plates were blocked with 2% BSA-PBS after washing. Washed plates were incubated with diluted serum samples, mouse IgE (BD Biosciences), anti-DNP-IgG2a, or anti-DNP-IgG1 (Accurate Antibodies, Westbury, NY) overnight at 4°C and later developed by using biotinylated anti-IgE, IgG2a or IgG1 detection antibodies (BD Biosciences), avidin-peroxide, and ABTS substrate (KPL, St Paul, MN).

### Flow cytometry analysis of IgE producing plasma cells

Mice were sacrificed at week 78 of the protocol and single-cell suspensions of splenocytes were prepared in ice cold staining buffer (PBS including 0.5 mM EDTA, 0.05 mM sodium azide, 0.5% BSA). First, surface staining with unlabeled anti-IgE (to block membrane IgE), APC anti-CD138, BV711-anti-CD3, and anti-CD16/32 (Fc-block) (all from BD Biosciences), CA was performed. Live-dead discriminating dye (Live-Dead Aqua, Invitrogen, CA) was included. Cells were washed and incubated with fixation/permeabilization buffer (BD Biosciences, CA) for 15 mins, washed with permeabilization buffer (BD Biosciences, CA), and then incubated with FITC-anti-IgE, in permeabilization buffer. After washing, cells were treated with Cytofix buffer (BD Biosciences, CA) for 15 mins for post-fixation, washed, and then data were acquired on an LSRII flow cytometer (Becton Dickinson, CA). Flow cytometry analysis was performed using Flow Jo (Tree Star, CA) as follows. Live singlet cells were then analyzed for IgE^+^ plasma cells (FITC-IgE +; APC-CD138+ cells).

### Safety testing of EBF-2 in a mouse model

To evaluate the safety of EBF-2, the sub-chronic toxicity assay was performed on C3H/HeJ mice as in our previous studies (52). Naïve C3H/HeJ mice were fed 40 mg/mL of EBF-2, which is 5 times the daily therapeutic dose, for 14 days. Sham (water) fed mice served as controls (sham). Blood samples were collected at the end of the experiment. Liver and kidney function and complete blood count (CBC) were performed by ALX laboratories, NY.

### High performance liquid chromatography fingerprint analysis of EBF-2 and ex vivo detecting EBF-2 active compound by liquid chromatography–mass spectrometry

HPLC analysis was performed on a Waters 2690 HPLC system coupled with a 2996 PDA detector (Waters, Milford, MA) for each batch of FAHF-2 and EBF-2 using the method described previously (52). Each sample was first dissolved in 2 mL of the mixture of mobile phases at a 1:1 ratio and centrifuged at 10,000 rpm for 10 mins. The sample amount injected for HPLC fingerprint analysis was based on the human daily dose used (1:200 of human daily dose). FAHF-2’s human daily dosage is 19.8g, therefore the concentration of FAHF-2 injected was 99mg/mL. For EBF-2, the human daily dose is 4.58g, therefore the concentration of EBF-2 injected to HPLC was 22.9mg/mL. 10 µL of the supernatant was injected into the HPLC system and separated on a ZORBAX SB-C18 (5 µm, 150 mm x 4.6 mm, column (Agilent, Santa Clara, CA). Aqueous formic acid (0.1%) was used as mobile phase A, while acetonitrile (Fisher Scientific, NJ) served as mobile phase B. The separation was performed using a linear gradient elution of 2% to 25% mobile phase B in 45 min, 25% to 35% in the following 25 mins, 35% to 55% in the next 15 mins, and 55% to 75% in the final 10 mins. The flowrate was maintained at 1 mL/min. Data was collected and processed using Waters Empower software.

EBF-2 active compound in tissue samples were analyzed using LC-MS system. Briefly, tissue samples were cut into small pieces and soaked in methanol. The extracts were analyzed on an Agilent 1200 HPLC instrument with an Agilent Eclipse Plus-C18 column (5 μm, 250 mm × 4.6 mm), coupled with an Agilent 6130 Single Quad Mass Spectrometry. The samples were eluted with acetonitrile-water (5-95%) containing 0.1% formic acid (v/v) over 100 mins.

### Real time polymerase chain reaction

U266 cells (1.0 × 10^6^ cells/mL) were incubated with or without berberine for 3 days. Cells were harvested and total RNA was isolated using Trizol (Gibco BRL, Rockville, MD). The RNA concentrations were quantified by triplicate optical density (OD) readings (Bio-Rad SmartSpect 3000; Bio-Rad, Hercules, CA). Reverse transcription was performed to yield cDNA using ImProm-II™ Reverse Transcriptase (Promega Corporation, Madison, WI) as per the manufacturer’s instructions. The RT-PCR amplification was performed using Maxima™ SYBR Green qPCR Master Mix (2X) kit (Fisher Scientific, Pittsburgh, PA). Primer sequences of XBP1, BLIMP1, STAT-6, BCL-6 and GAPDH were from previously published literature and listed in **Supplemental Table 2** (55, 60–62).

### Seahorse mitochondrial stress assay

To determine the effect of EBF-2’s active compound berberine on mitochondrial metabolism of an IgE producing plasma cell line, we used the Seahorse mitochondrial stress assay. XF cell Mito stress test kits were obtained from Agilent (Santa Clara, CA). Each well of the XF24 cell culture plates was coated with 50 µL of Corning Cell-Tak cell and tissue adhesive at a density of 3.5 µg/cm^2^ for 20 mins followed by washing with 200 µL water and 20 min of drying. Fresh assay medium was prepared by supplementing 2 mM glutamine into XF cell base medium, DMEM with an adjusted pH of 7.4. Next, 1 × 10^5^ of U266 cells resuspended in 100 µL of assay medium were seeded into each well of the coated plate by centrifugation in a swing-bucket rotor at 450 rpm for 1 min without braking. After reversing the orientation of the plates, they were centrifuged again at 650 rpm for 1 min without braking. Plates were transferred to a 37°C incubator not supplemented with CO_2_ and incubated for 25-30 mins. Then, 500 µL of warm assay medium, containing DMSO or various concentrations of BBR was slowly and gently added into the wells. After a 15 mins CO_2_-free incubation, the cells were ready for the assay on a Seahorse XFe24 Analyzer. Oligomycin (3 µM final concentration), carbonyl cyanide-4 (trifluoromethoxy) phenylhydrazone (FCCP, 3 µM final concentration) and rotenone and antimycin (3 µM and 1 µM final concentration, respectively) were diluted in the assay medium and loaded into ports A, B, and C of the XF24 assay plate. The machine was calibrated, and the assay was performed using the Mito stress test assay protocol per the manufacturer’s recommendations. The extracellular acidification rate (ECAR) and oxygen consumption rate (OCR) were measured under basal conditions after sequential addition of the above-mentioned drugs.

### Statistical analysis

All statistical analyses were performed using GraphPad Prism 9 (San Diego, CA). One-way ANOVA (analysis of variance) was performed followed by Bonferroni correction for all pairwise comparisons. For skewed data, differences between groups were analyzed by one-way ANOVA on ranks followed by Dunn’s method for all pairwise comparisons. Data for symptom score correlation with IgE were analyzed using Spearman correlation. Pearson correlation was used for all other correlation analyses. *p*-value calculations were two-tailed and a *p* value < 0.05 was considered as statistically significant.

## Results

### EBF-2 dose-dependently inhibited IgE production in an IgE plasma cell line

As compared to the untreated cells, EBF-2 significantly decreased IgE production beginning at 1.9 μg/mL (*p*<0.05) with complete inhibition of IgE production at 60 μg/mL *(p*<0.001) (**Figure 1A**). The IC_50_ value was 4.70 ± 1.16 μg/mL (**Figure 1B**). There was no observed cytotoxicity at any tested concentrations (**Figure 1C**). The parent formula FAHF-2 also significantly decreased IgE production at 62.5 μg/mL (*p*<0.01) with complete inhibition of IgE production at 500 μg/mL(*p*<0.001) (**Figure S1A**). The IC_50_ value of the parent formula FAHF-2 was 79.7 ± 17.39 μg/mL (**Figure S1B**), with no cytotoxic effect across all concentration (**Figure S1C**). Furthermore, we analyzed the effect of three different batches of FAHF-2 (F2-1106, F2-0202, F2-0909) (**Figures S2A–C**) and EBF-2 (EBF-2-0303, EBF-2-0808, EBF-2-0130) (**Figures S2D–F**) on IgE production by U266 cells respectively and found consistent results between different batches. Taken together, EBF-2 is markedly more inhibitory on IgE production than its parent formula while retaining high cellular safety and batch to batch consistency

### EBF-2 treatment suppressed peanut anaphylaxis associated with suppression of peanut specific-IgE without affecting IgG1 or IgG2a production

**Figure 2:**
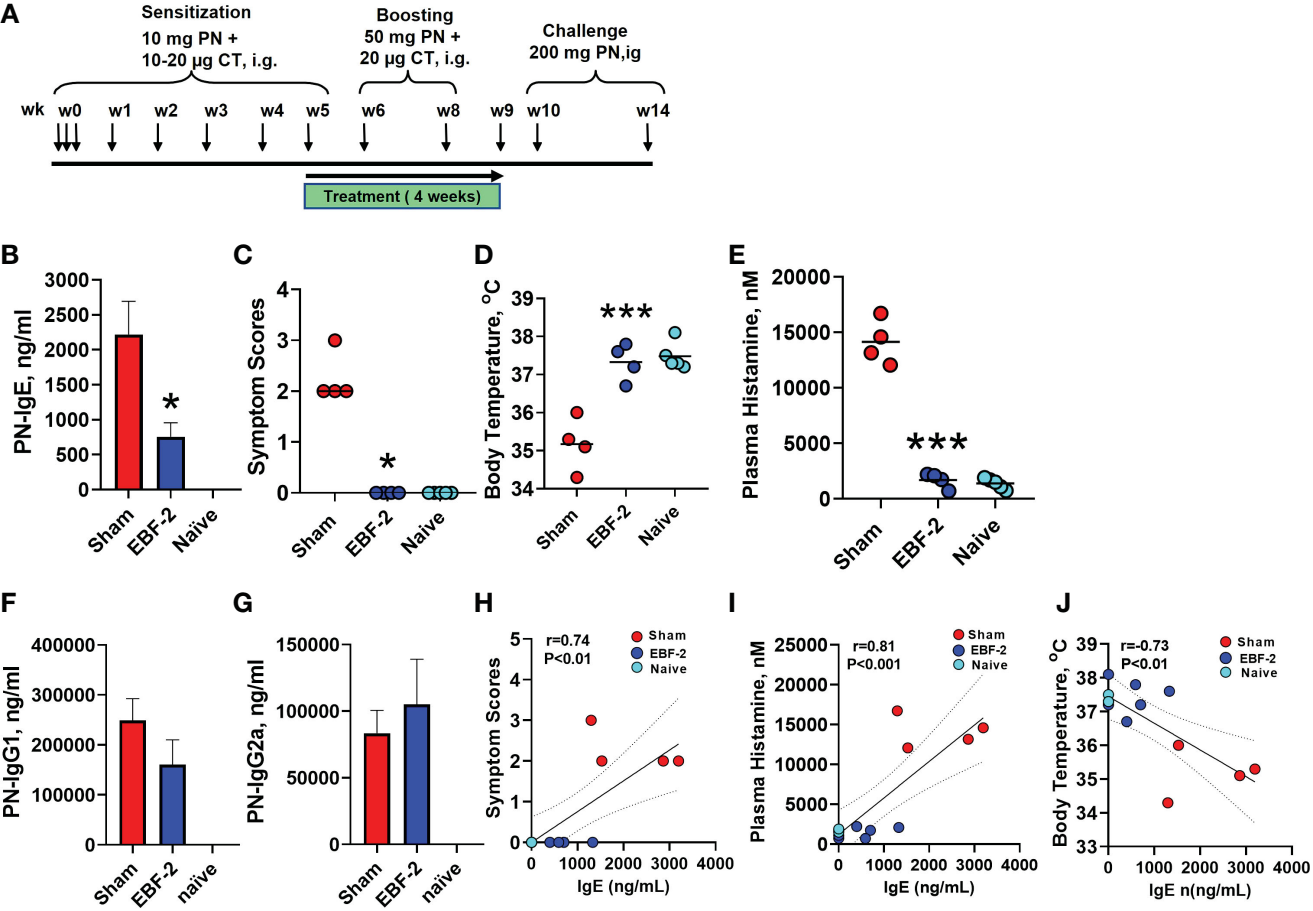
Effect of EBF-2 in a peanut allergic mouse model. **(A)** Experimental design for sensitization, treatment, and challenge: 6 weeks old C3H/HeJ mice were orally sensitized with 10 mg PN and 10-20 µg cholera toxin at weeks 0 through 5. Mice were boosted with 50 mg PN and 20 µg cholera toxin at weeks 6 and 8. Daily oral EBF-2 treatment (7.68mg/mouse/day) started at week 5 and continued for four weeks. Mice underwent oral PN challenges (200mg) at week 10 and week 14. **(B)** PN specific IgE measured by ELISA at week 14. **(C)** Symptom scores; **(D)** Body temperatures and **(E)** Plasma histamine levels 30 mints following oral PN challenge at week 14. **(F)** PN-specific IgG1; and **(G)** PN-specific IgG2a were measured by ELISA. **(H)** Spearman correlation between PN-IgE and symptom scores, **(J)** Pearson correlation between PN-IgE and Body temperature. **(I)** Pearson correlation between PN-IgE and plasma histamine Bars indicate group means. *P < 0.05; ***P < 0.001 vs. Sham. N=4-5 mice/group.

We next determined EBF-2’s inhibitory effect on IgE levels and its protective effect against peanut anaphylaxis in a murine model (**Figure 2A**). Four weeks after discontinuation of EBF-2 treatment (at week 14 following initial PN sensitization), peanut (PN)-specific IgE levels were significantly reduced by approximately 70% in EBF-2 treated mice compared to sham treated mice (**Figure 2B**, *p*<0.05 vs. Sham). Following intragastric challenge, all sham-treated mice developed anaphylactic symptoms, with symptom severity scores ranging from 2-3. In sharp contrast, EBF-2 treated mice were completely protected from anaphylaxis (**Figure 2C**, EBF-2 vs. Sham: median score 0 vs. 2, *p*<0.05). Hypothermia, a decrease in core body temperature, is a symptom of anaphylaxis in mice. We measured rectal temperatures every 30 minutes after the intragastric challenge, EBF-2 prevented hypothermia, the mean post-challenge body temperature in EBF-2 group was significantly higher than the Sham group and not different from naïve mice (**Figure 2D**, sham vs. EBF-2 vs. naïve: 35.18±0.3°C vs. 37.33±0.2°C vs. 37.33±0.2°C, *p*<0.001 vs. sham). Anaphylaxis is associated with an increase in plasma histamine levels and plasma histamine levels in EBF-2 mice were markedly and significantly lower than in sham mice (**Figure 2E** Sham vs. EBF-2 mean±SEM: 14,134±1004 nM vs. 1689±340 nM). The EBF-2 treated group’s plasma histamine level was not significantly different from the normal range of histamine levels in the naïve group (1392±213 nM). PN-specific IgG1 and PN-specific IgG2a production were not affected (**Figures 2F, G**). In this model, symptom severity and plasma histamine levels strongly correlated with IgE levels (**Figures 2H, I**, r=0.74, *p <*0.01; r=0.81, *p <*0.001, respectively), whereas body temperatures at challenge were inversely correlated with IgE (**Figure 2J**, r=-0.73, *p <*0.01).

### EBF-2 treatment produced long term protection from peanut anaphylaxis and reduced IgE^+^ plasma cell numbers

**Figure 3:**
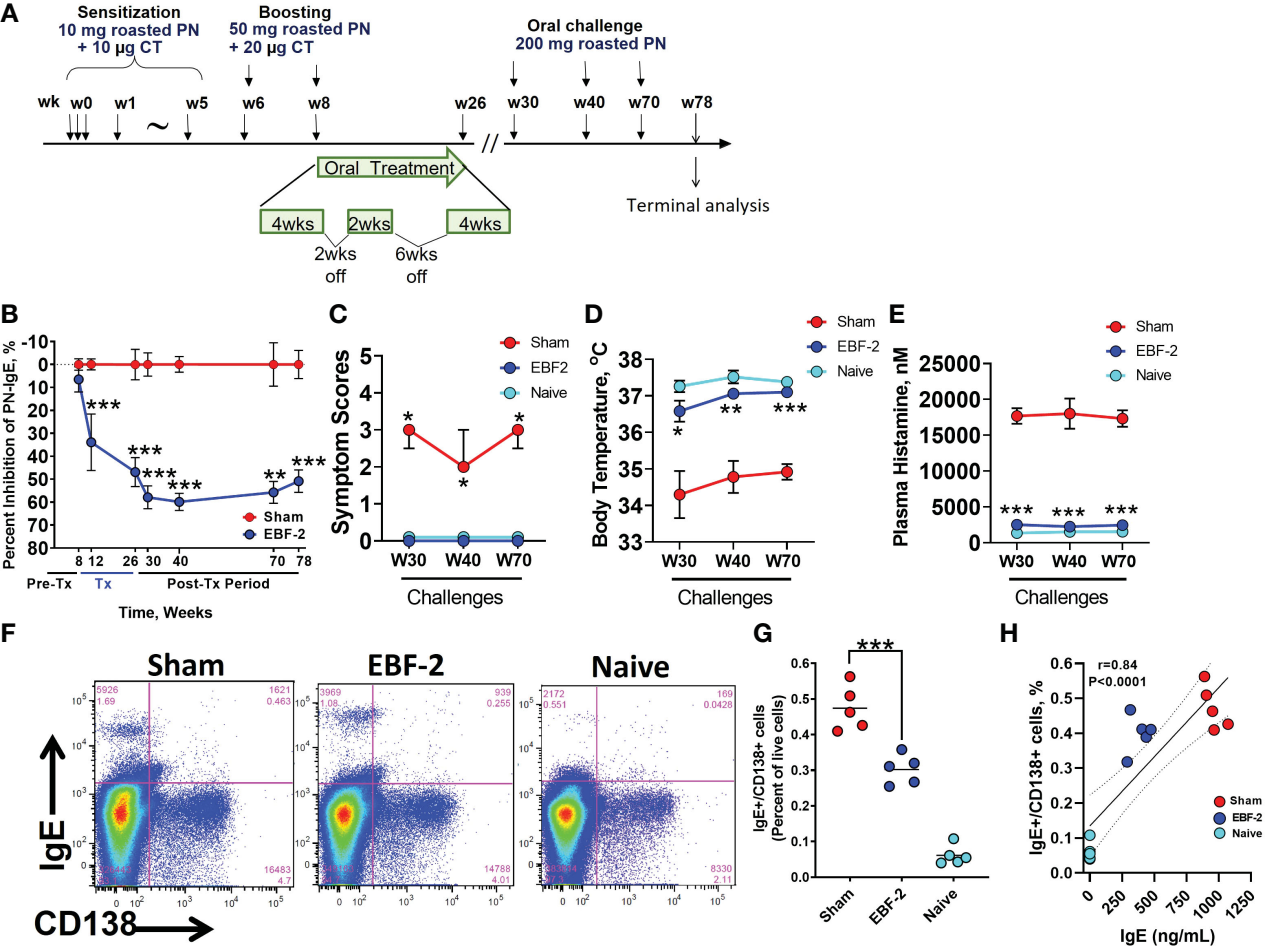
Long term effect of EBF-2 on peanut-specific IgE, anaphylaxis and IgE^+^PC. **(A)** Experimental protocol. **(B)** Effect of EBF-2 on percent inhibition of PN-specific IgE over duration of the experiment. **(C–E)** symptoms scores, body temperature and plasma histamine levels 30 minutes following challenges at weeks 30, 40 and 70. **(F)** Representative flow cytometry panels showing the percentage of IgE+/CD138+ PCs (upper right quadrant) in spleens of mice in sham, EBF-2, and naïve groups. **(G)** Scatter graph showing data for individual mice across experimental groups shown in **(F)** Bars are group means. **(H)** Correlation between IgE+/CD138+ plasma cells and PN-specific IgE levels in mice across all experimental groups. r value is the Pearson coefficient of correlation. *p<0.05, **p<0.01, ***p<0.001 vs sham vs. sham. N=5 mice/group. PC, plasma cells.

We investigated the long-term protection by EBF-2 and its effect on long-lived IgE producing plasma cells in a peanut anaphylaxis murine model (**Figure 3A**). EBF-2 significantly reduced PN-specific IgE during therapy (weeks 8-26) (*p*<0.001) and maintained consistent levels even after completion of the treatment, up to week 78 (*p*<0.01, *p*<0.001) (**Figure 3B**). Post-treatment challenges conducted at week 30, week 40 and week 70, respectively, showed that EBF-2 treated mice were completely protected from anaphylaxis (**Figure 3C**, EBF-2 vs Sham, *p*<0.05), prevented hypothermia (**Figure 3D**, *p*<0.001), and reduced plasma histamine levels (**Figure 3E**, *p*<0.001). Furthermore, EBF-2 treated mice showed significantly lower numbers of the IgE^+^/CD138+ plasma cells from the spleen compared to Sham treated mice (**Figures 3F, G**, *p*<0.001). There was a significant correlation between IgE+/CD138+ plasma cells and PN-specific IgE levels (**Figure 3H**, r=0.84, *p*< 0.0001). These data highlight the persistent protection of EBF-2 from anaphylaxis following peanut exposure.

### EBF-2 formula had a high safety profile

EBF-2’s safety was evaluated with a sub-chronic toxicity protocol. C3H/HeJ mice were fed 5 times the normal daily dose of EBF-2 for 14 consecutive days and observed for 2 weeks. No morbidity or mortality was observed. Serum ALT and BUN levels of EBF2 and sham-treated mice were all within normal range (**Table 1**). CBC results in the EBF-2 treated group were similar to those of sham treated mice and were all within the normal range. Thus, the EBF-2 formula has a high safety profile.

**Table 1:** *In vivo* sub-chronic safety assessment of EBF-2.

	Treatment (5x daily dose)		Reference range
	Water (n=5)	EBF-2 (n=5)	
**Morbidity**	0/10	0/10	NA
**Mortality**	0/10	0/10	NA
**ALT (U/L)**	32.6±12.4	55.8±27.7	28-129
**BUN (mg/dL)**	18.8±3.5	23.2±1.9	7.0-28
**WBC (10^3^/µL)**	5.1±1.1	6.2±1.8	3.9-13.9
**RBC (10^3^/µL)**	10.4±0.7	9.9±0.9	7.14-12.2
**Hb (g/dL)**	13.3±1.1	12.4±1.2	10.8-19.2
**PLT (10^3^/µL)**	645.6±243.2	923.6±483.2	565-2159
**Neu (10^3^/µL)**	1.3±0.3	1.7±0.6	0.42-3.09
**L (10^3^/µL)**	3.3±0.9	3.4±0.9	2.88-11.15
**Eos (10^3^/µL)**	0.1±0.0	0.1±0.0	0.01-0.50
**Bas (10^3^/µL)**	0.0±0.0	0.0±0.0	0-0.14

Naive mice were fed a therapeutic dose 5 times daily for 14 days. Sham fed mice served as controls (sham). Blood samples were collected after termination of experiments. Blood urea nitrogen (BUN) and alanine aminotransferase (ALT) measurements for evaluation of kidney and liver functions, respectively, and complete blood count (CBC) testing were performed. ALT, Alanine Aminotransferase; BUN, Blood Urea Nitrogen; WBC, White Blood Cells; RBC, Red Blood Cells; Hb, Hemoglobin; PLT, Platelets; Neu, Neutrophils; L, Lymphocytes; Eos, Eosinophils; Bas, Basophils; NA, Not available.

### HPLC fingerprints reveal a higher berberine peak in EBF-2 than FAHF-2 and detecting BBR ex vivo after feeding a single dose of EBF-2 by LC-MS

**Figure 4:**
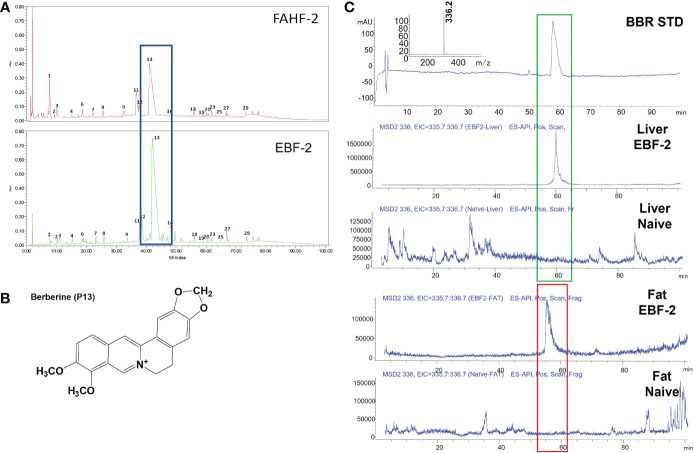
Characterization of FAHF-2 and EBF-2 products. **(A)** HPLC fingerprint of FAHF-2 and EBF-2. The x-axis indicates retention time in minutes, while the y-axis indicates an absorbance unit (AU). **(B)** Chemical structure of Berberine. **(C)** The presence of berberine in the liver and fat tissue samples of mice treated with E-B-FAHF-2. Mice were fed with the EBF-2 formula, and tissue samples were collected 5 days after oral administration. Mass spectra of the berberine standard; m/z: 336.2 was detected. Berberine presented in the liver of EBF-2 treated mice. No berberine was detected in liver samples of naïve mice. Berberine presented in fat tissue samples of EBF-2 treated mice. No berberine was detected in fat tissue samples of naïve mice. The illustrations are representative of 3-5 samples.

We previously showed that berberine (BBR) isolated from FAHF-2 and B-FAHF-2 reduced IgE by plasma cell lines and human PBMCs from food allergic patients (55), and demonstrated that the concentration of BBR can be a pharmacological marker of FAHF-2 and EBF-2. We therefore determined the concentration of BBR in EBF-2 and compared with parent formula FAHF-2 (**Figure 4A**). A total of 29 peaks (P) were detected in FAHF-2 and EBF-2 batches. The major peak 13 (P13) was identified as BBR (**Figure 4B**). The peak area of BBR (Mean ± SED) in EBF-2 was significantly higher than that in FAHF-2 (62.83 ± 3.53% vs 33.26 ± 6.40% overall total peaks *p*<0.05 (**Supplemental Table 3**). We have identified other peaks such as Magnoflorine (P6), Phellodendrine (P8), Jatrorrhizine (P12), Ganolucidic acid D (P18), and Ganoderic acid H (P27), but the differences between these peaks were not statistically significant. After the purification process, the constituents (less polar small molecules) in EBF-2 were more concentrated. We also calculated the BBR concentration by using the equation collected from the peak area of BBR standard versus the concentration (BBR concentration (µg/mL) = BBR pear area/1000/72.23). The BBR in FAHF-2 was calculated as 0.36%, while the EBF-2 contains 4.4% of BBR. The BBR concentration was approximately 12 times higher in EBF2 than in FAHF-2 (data not shown). We detected BBR in liver and fat tissue 5 days after oral administration of EBF-2 by LC-MS and demonstrated that BBR is a major bioavailable compound within EBF-2 when compared to naïve mice (**Figure 4C**).

### EBF-2 bioactive compound BBR inhibited IgE production and transcription factor XBP1, BLIMP1, and STAT6, and increased BCL-6 by IgE producing plasma cell

**Figure 5:**
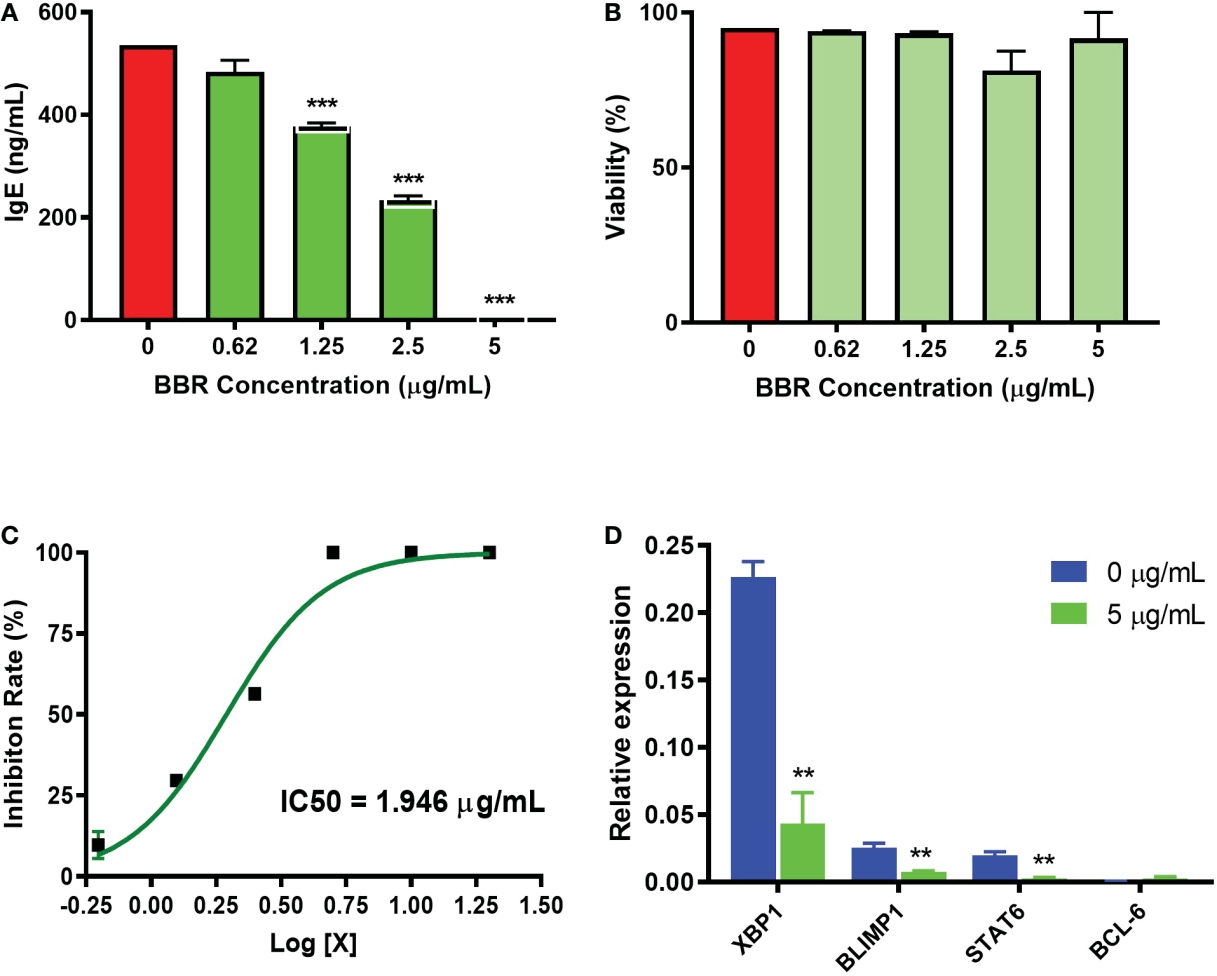
Effect of BBR on IgE production and transcription factor gene expression in U266 cells. **(A)** BBR dose-dependently inhibited the IgE production by U266 cells. **(B)** Cell viability of BBR on U266 cells. **(C)**. C_50_ value of BBR on IgE production was 1.946 μg/mL. **(D)** The relative expression level of XBP1, STAT6, BLIMP1, and BCL-6 genes vs. GAPDH. **p<0.001; ***p<0.001 vs. untreated. N=3 independent culture.

We evaluated the active compound BBR identified from EBF-2 and determined its effect on the regulation of IgE plasma cells at the transcriptional level, *in vitro* using U266 cells. BBR dose-dependently inhibited IgE production approaching 100% inhibition at 5 µg/mL (**Figure 5A**), without any cytotoxicity across the doses (0.625 – 5 μg/mL) **(** **Figure 5B**) with an IC_50_ value 1.946 μg/mL (**Figure 5C**). We evaluated BBR’s effects on the gene expression of XBP1, BLIMP1 and STAT-6, which are genes that have been shown to be upregulated during plasma cell activation. BBR significantly inhibits the gene expression of XBP1, BLIMP1 and STAT6 compared to untreated cells (p <0.01, **Figure 5D** **)**. BCL-6 reportedly inhibits long-lived plasma cell survival, and it has been shown to be upregulated in plasma cell activation. Therefore, we measured the effect of BBR on the gene expression of BCL-6, however our results showed that the increase expression of BCL-6 gene was not statistically significant. Taken together, we showed that the suppression of IgE in IgE^+^ plasma B cells by BBR is mediated by down-regulation of XBP1 and BLIMP1.

### Berberine, a major active compound in EBF-2, inhibited IgE producing plasma cell mitochondrial metabolism

**Figure 6:**
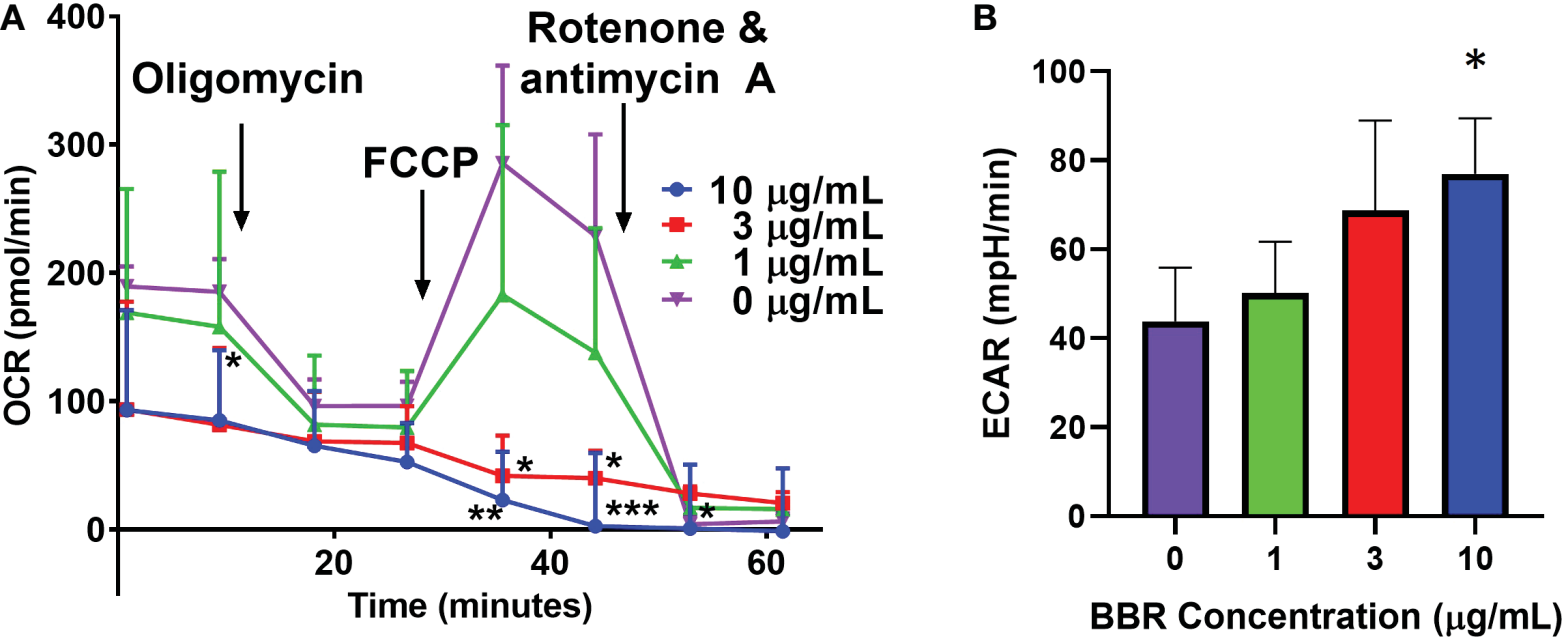
Effect of BBR on mitochondrial respiration rate in U266 cells. **(A)** U266 cells were pre-treated with DMSO or the indicated concentration of BBR for 15 min. Oligomycin (3 μM, inhibitor of mitochondrial complex V), FCCP (4 μM, stimulator of mitochondrial complex IV), and combination of rotenone (3 μM) and antimycin A (1 μM) (inhibitors of mitochondrial complex I and III, respectively) were injected at the indicated time per manufacturer instructions. The oxygen consumption rate (OCR) was recorded over time. **(B)** BBR dose-dependently increased basal glycolytic rate 15 min after treatment. Data represents triplicate cultures and is expressed as mean ± SEM. *p <0.05; **P<0.01; ***p<0.001 vs. untreated.

Plasma cells have a nutrient uptake and energy demand for the production and secretion of antibodies compared to their counterparts of B cells and plasmablasts (44). Emerging evidence suggests that glucose availability and energy metabolism are important for regulating plasma cell antibody production, secretion and survival at post-transcriptional levels (44, 63). Traditionally, BBR has been used as a glucose lowering agent in Type II diabetes through inhibition of mitochondrial respiratory complex I (64–67). We next asked whether BBR disrupts energy metabolism and changes glucose utilization in IgE plasma cell line U266 by a seahorse Mito stress assay. The assay measures mitochondrial respiration and function glycolysis by directly measuring OCR (Oxygen Consumption Rate) and ECAR (Extracellular Acidification Rate). Vehicle treated U266 cells were used to establish some key parameters of mitochondrial function (**Figure 6A**) including basal respiration, proton leak (after oligomycin injection, which inhibits ATP production through complex V), maximal respiration capacity (after injecting FCCP, which uncouples ATP production from electron transport), and non-mitochondrial respiration (after injecting complex I and III inhibitors rotenone and antimycin A). Interestingly, BBR pretreatment significantly suppressed basal OCR (p<0.05) and FCCP-induced maximal OCR (p<0.001) in a dose-dependent manners, with almost complete inhibition of mitochondrial respiration at 3 µg/mL and 10 µg/mL, respectively. To compensate for energy crisis resulted from mitochondrial respiratory inhibition, BBR-treated U266 cells increased the rate of glycolysis to increase ATP production from glycolysis pathway, as indicated by significantly elevated ECAR (**Figure 6B**, BBR at 10 µg/mL vs. untreated cells, p<0.05). These results suggest BBR inhibits mitochondrial respiration in IgE^+^PCs, which lead to cellular energy crisis and decreased the availability of glucose molecules for other pathways such as IgE Ab glycosylation.

## Discussion

IgE plays a key pathogenic role in FA-related hypersensitivity. Enthusiasm about IgE regulatory interventions for FA therapy remains high yet, interventions to modulate IgE^+^PCs remain undeveloped. We tested direct effects of EBF-2, a refinement of parent formula FAHF-2, on IgE production using U266 cells, a well-established IgE^+^ plasma cell line. We demonstrated the consistent inhibition of IgE production with EBF-2 across 3 separate batches, without cytotoxicity, and more potently than parent FAHF-2. EBF-2 was 16-fold more potent than parent formula FAHF-2, suggesting a superior effect compared to the parent formula. Using a PN allergic murine model, we demonstrated that EBF-2 decreased PN-specific IgE levels by ~70% following 4 weeks of oral treatment compared to sham-treated mice in an early EBF-2 treatment protocol, with no significant changes in PN-specific IgG1 or IgG2a levels and demonstrated a high safety profile. EBF-2 significantly protected PN allergic mice from anaphylaxis with an 8.5-fold lower daily dose than parent FAHF-2 (68), highlighting its efficacy and potency. The mechanism underlying this persistent therapeutic effect is unknown but may be due to suppression of long-lived IgE^+^PCs. Using a PN allergy model, we showed that the percentages of IgE^+^PCs, largely LLPCs at this 8-week post antigen exposure timepoint, were significantly reduced in EBF-2 treated mice and correlated with the peanut-IgE levels. This is important because these IgE^+^PCs, which are LLPCs known to resist immunosuppressive or ablative therapies (24, 25, 69, 70), showed significant reduction following EBF-2 treatment. This indicates that EBF-2 may have a potential to alter the process of persistent peanut allergy, but this requires further investigation.

As a first attempt to understand EBF-2 suppression of IgE^+^PCs and given that BBR is found at higher levels in EBF-2 than in FAHF-2 by HPLC, we identified BBR as a bioavailable compound within EBF-2 by LC-MS analysis following oral feeding. This provides a rationale to study BBR as a bioavailable active compound to regulate IgE production. We showed that BBR suppressed IgE production by a human IgE producing plasma cell line in a dose-dependent manner. Since no cytotoxicity was observed even at the dose (5 μg/mL) which BBR eliminated IgE production, we hypothesized it may act on mechanisms controlling IgE production and secretion. IgE^+^PCs are under constant stress from antibody production and secretion. XBP1, a transcription factor, promotes and maintains plasma cells antibody production and secretion under ER stress as a compensating response (44, 63). We found a significant reduction of XBP1 gene expression in BBR-treated cells (~ 5-fold reduction) at a non-toxic dose compared to untreated cells. We also found that BBR inhibited BLIMP1, which promotes plasma cell survival (45). In addition, we found that STAT-6 was significantly reduced in a BBR treated IgE^+^ plasma cell. STAT-6 is reported to be mainly involved in cooperating on IL-4-induced up-regulation of an IgE germline promoter (71). However, the role of STAT-6 on terminally differentiated IgE^+^PCs has not been reported, requiring further investigation. In contrast, BCL-6, a transcriptional repressor of IgE production (72), tended to be increased. These findings suggest that BBR modulation of XBP1 and other transcription factors may together down-regulate IgE^+^PCs.

**Figure 7:**
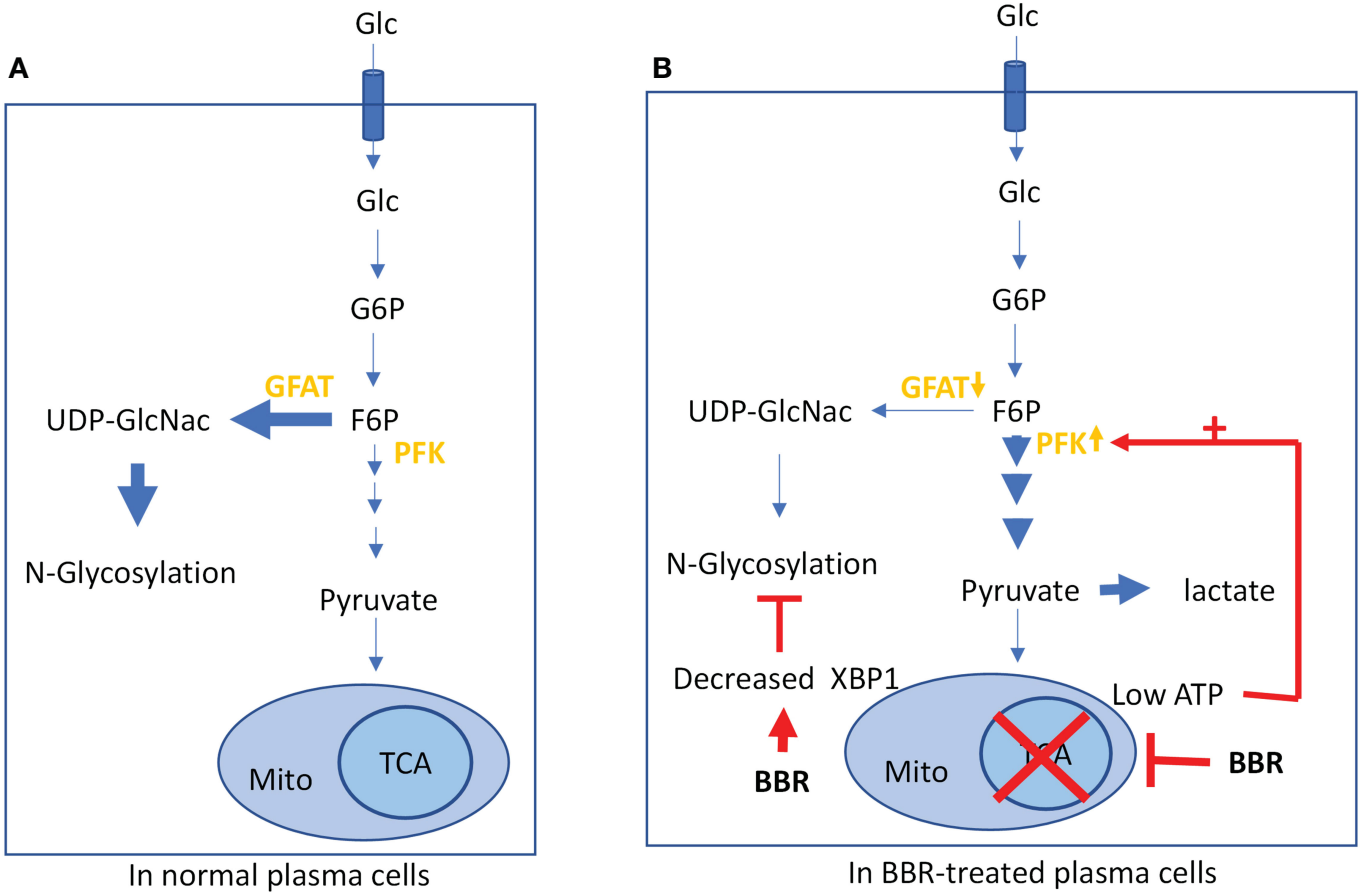
Role of BBR in regulating energy consumption: **(A)** Glucose metabolism in normal plasma cell to produce ATP *via* TCA cycle. **(B)** BBR treatment decreases mitochondrial respiration, suppresses XBP1, and inhibits glycosylation.

In addition to transcriptional regulation, energy metabolism has emerged as an important regulator of plasma cell survival and function (44). Previous studies have shown that long-lived plasma cells (LLPCs) used 90% of glucose to glycosylate antibodies; however, when these cells were under energy stress such as challenged with a mitochondrial inhibitor, they diverted glucose to glycolysis to form pyruvate to support energy production, which was accompanied by a marked decrease in antibody secretion (73). Classically, BBR has been used to treat type II diabetes (74). Suppressing mitochondrial metabolism and promoting glycolysis have been suggested as mechanisms underlying BBR’s anti-diabetic effects (66). Here we demonstrate, for the first time, that BBR reduces IgE plasma cell mitochondrial metabolism. We believe that BBR inhibits IgE^+^PC mitochondrial respiration likely forcing IgE^+^PCs to produce ATP through upregulated glycolysis. The metabolic decision point for glucose between glycolysis and hexosamine biosynthesis for glycosylation occurs at fructose-6-phosphate, catalyzed by phosphofructokinase (PFK). It is established that a low cellular ATP level allosterically activates the enzyme PFK, one of the rate-limiting enzymes in glycolysis, thus leading to the increase in glycolysis and the subsequent decrease in hexosamine biosynthesis for glycosylation (**Figure 7A**). Therefore, we feel that for the scope of this paper, showing the >50% increased glycolytic rate after BBR treatment highly likely led to the diversion of glucose away from IgE glycosylation (**Figure 7B**), potentially causing accumulation of immature (unglycosylated) IgE in the ER and in turn triggering ER stress. Under normal circumstances this triggers up-regulation of XBP1 to compensate for energy depletion. However, this will not happen in the presence of BBR that suppresses XBP1, leading to a cellular energy crisis (**Figure 7B**). Thus, BBR switches off IgE production from IgE^+^PCs by regulating both transcription factors and mitochondrial metabolism.

In this study, our goal is to test the potency of a refined botanical medicine in murine model of peanut allergy, we therefore orally sensitized using Th2 adjuvant and orally challenged female C3H/HeJ mice. The advantage is that this murine model showed persistent peanut allergy, which allow us to test the durability of EBF-2 on food allergy and study long-lived IgE producing plasma cells. The reason to use female mice is to consider that females are more susceptible to food allergies (75) and have been widely used in food anaphylaxis studies (75–83). Therefore, we intended to use established model. Additional limitation of this study is lack of positive treatment control. At present, there is limited or no peanut therapy showing sustainable protection. Our previous publication showed that the effect of protection again anaphylaxis by peanut oral immunotherapy (OIT) is transient. At 5 weeks post therapy, the reactions returned by 90% of OIT treated mice following peanut challenge (51). Therefore, we included peanut allergic mice treated with water as sham treatment control (equivalent to placebo control in human trials. Since BBR has poor bioavailability, we further are working to develop a technology to encapsulate BBR with nano particle to prevent degradation and enhance absorption in the gastric tract. In future, we intend to investigate nano-BBR intervention on transcription factors and mitochondrial metabolism in murine PN allergic models. Furthermore, in order to conform the direct evidence of BBR effect on IgE glycosylation, glycoproteomic analyses should be considered in future.

In conclusion, this study demonstrated that a novel botanical medicine, EBF-2, significantly and consistently suppressed IgE production and is markedly more potent than its parent formula FAHF-2. EBF-2 significantly suppressed PN specific IgE production with complete protection against anaphylaxis and long-lasting effects associated with suppression of IgE^+^PCs in a murine model. The mechanism of BBR, the active EBF-2 compound in suppressing IgE may be partially associated with its inhibitory effect on XBP1 and mitochondrial metabolism leading to insufficient energy and transcriptional activation for IgE IgH/IgL synthesis and antibody glycosylation. Further understanding of how EBF-2 and BBR regulate established IgE production by IgE^+^ PCs may lead to new interventions to target key mechanisms of IgE-mediated food allergy.

## Data availability statement

The original contributions presented in the study are included in the article/**Supplementary Material**. Further inquiries can be directed to the corresponding authors.

## Ethics statement

The animal study was reviewed and approved by Icahn School of Medicine at Mount Sinai.

## Author contributions

NY, ARM, KS, MK, ZW, IM, YS, OF, and YG were significantly involved in conducting experiments, data analysis, and manuscript preparation. JW, DC, DD, and HS were significantly involved in manuscript revision. JZ, MM, and X-ML were significantly involved in study design, data interpretation, and manuscript revision. All authors contributed to the article and approved the submitted version.
