# Supplementary material for: Inhibition of pathologic immunoglobulin E in food allergy by EBF-2 and active compound berberine associated with immunometabolism regulation
Source: Front Immunol. 2023 Feb 7;14:1081121. doi: 10.3389/fimmu.2023.1081121 (PMC9941740; doi:10.3389/fimmu.2023.1081121)
Supplement: Supplementary file 1 [file Presentation_1.pptx]

## Slide 1
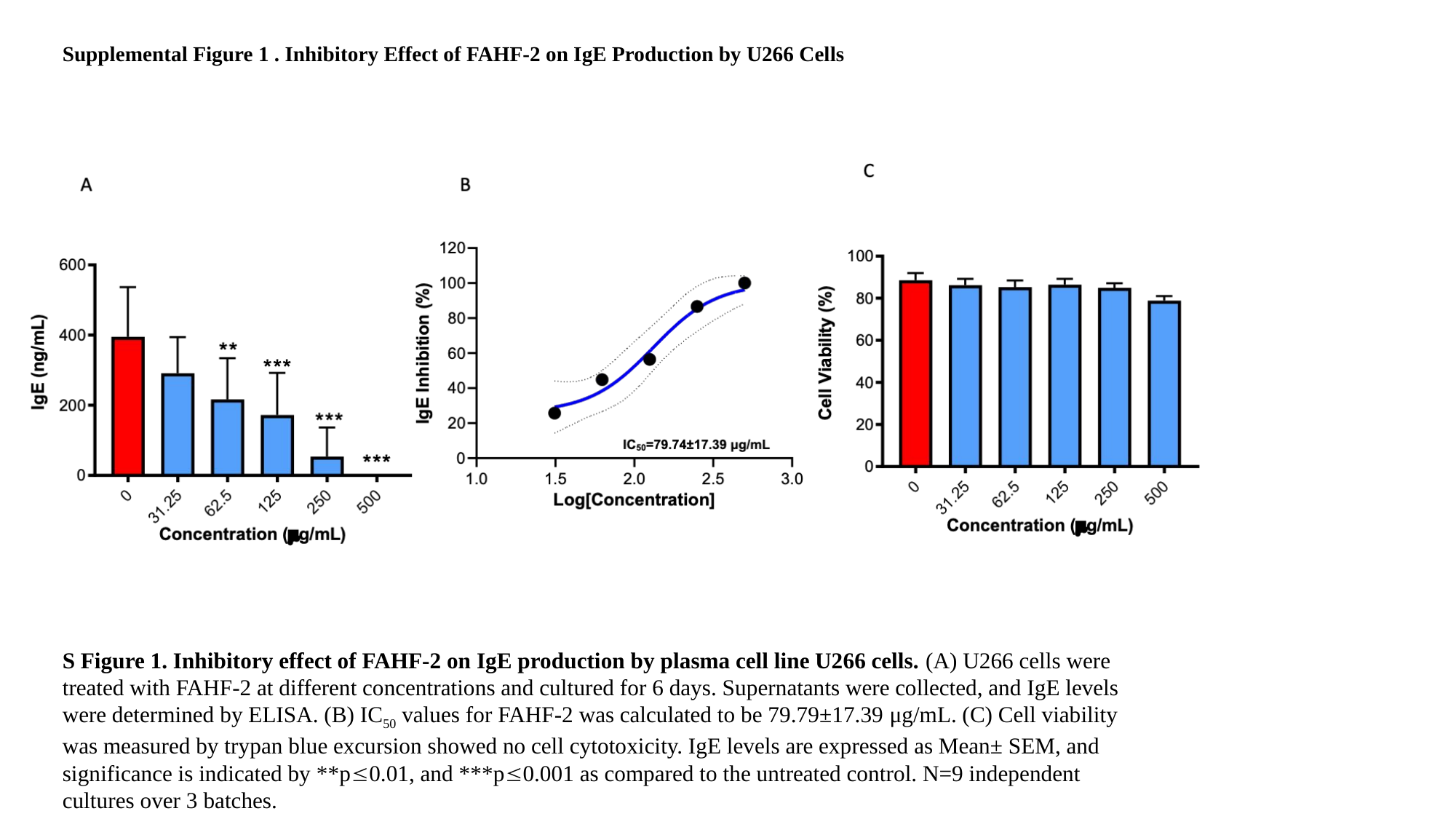

Supplemental Figure 1 . Inhibitory Effect of FAHF-2 on IgE Production by U266 Cells
S Figure 1. Inhibitory effect of FAHF-2 on IgE production by plasma cell line U266 cells. (A) U266 cells were treated with FAHF-2 at different concentrations and cultured for 6 days. Supernatants were collected, and IgE levels were determined by ELISA. (B) IC50 values for FAHF-2 was calculated to be 79.79±17.39 μg/mL. (C) Cell viability was measured by trypan blue excursion showed no cell cytotoxicity. IgE levels are expressed as Mean± SEM, and significance is indicated by **p0.01, and ***p≤0.001 as compared to the untreated control. N=9 independent cultures over 3 batches.

## Slide 2
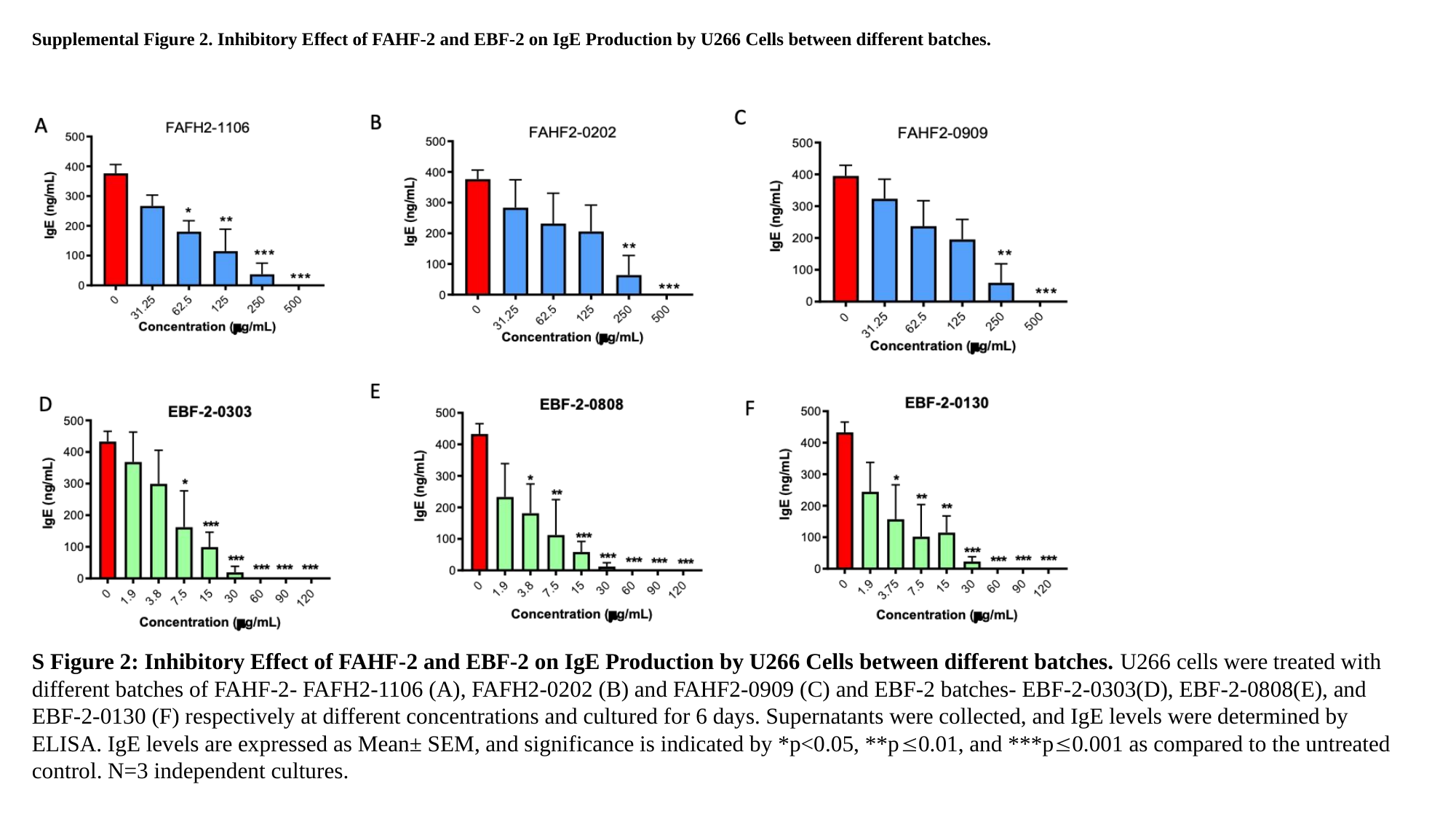

Supplemental Figure 2. Inhibitory Effect of FAHF-2 and EBF-2 on IgE Production by U266 Cells between different batches.
S Figure 2: Inhibitory Effect of FAHF-2 and EBF-2 on IgE Production by U266 Cells between different batches. U266 cells were treated with different batches of FAHF-2- FAFH2-1106 (A), FAFH2-0202 (B) and FAHF2-0909 (C) and EBF-2 batches- EBF-2-0303(D), EBF-2-0808(E), and EBF-2-0130 (F) respectively at different concentrations and cultured for 6 days. Supernatants were collected, and IgE levels were determined by ELISA. IgE levels are expressed as Mean± SEM, and significance is indicated by *p<0.05, **p0.01, and ***p≤0.001 as compared to the untreated control. N=3 independent cultures.
