# Supplementary material for: Inhibition of pathologic immunoglobulin E in food allergy by EBF-2 and active compound berberine associated with immunometabolism regulation
Source: Front Immunol. 2023 Feb 7;14:1081121. doi: 10.3389/fimmu.2023.1081121 (PMC9941740; doi:10.3389/fimmu.2023.1081121)
Supplement: Supplementary file 2 [file Table_1.docx]

**Supplemental table**

**Supplemental Table 1: FAHF-2 and EBF-2 batches.**

|  | **FAHF-2** | | | **EBF-2** | | |
| --- | --- | --- | --- | --- | --- | --- |
|  | Lot# | Manufacturing  Date | Shelf life  (As of 2020) | Lot# | Manufacturing  Date | Shelf life  (As of 2020) |
| Batch 1 | 110609 | Nov. 2009 | 11 years | 03032016 | Mar. 2016 | 4 years* |
| Batch 2 | 090910 | Sep. 2010 | 10 years | 08082017 | Aug. 2017 | 3 year* |

The manufacturing data and self-life of different batches of FAHF-2 and EBF-2 respectively.
