# Supplementary material for: Inhibition of pathologic immunoglobulin E in food allergy by EBF-2 and active compound berberine associated with immunometabolism regulation
Source: Front Immunol. 2023 Feb 7;14:1081121. doi: 10.3389/fimmu.2023.1081121 (PMC9941740; doi:10.3389/fimmu.2023.1081121)
Supplement: Supplementary file 3 [file Table_2.docx]

**Supplemental Table 2. Primer sequences of XBP1, BLIMP-1, BCL-6 and GAPDH.**

| Name | Sequences |
| --- | --- |
| Human STAT6-forward | 5′- GGCAGGGAATGGTAGTGGATAG-3′ |
| Human STAT6- reverse | 5′- TCAATCAGGGCCTCACCGTA-3' |
| Human Xbp1-forward | 5′- TCACCCCTCCAGAACATCTC-3′; |
| Human Xbp1-reverse | 5′- AAAGGGAGGCTGGTAAGGAA-3′; |
| Human BLIMBP-1-forward | 5′- ACCAAGGAATCTGCTTTTCAAGTATG-3′ |
| Human BLIMBP-1-reverse | 5′- CATCACTCCAATAACCTCTTCACTGT-3′ |
| Human BCL-6-forward | 5′- CTGGCTTTTGTGACG GAAAT-3′ |
| Human BCL-6-reverse | 5′- AACCTGAAAACCCACACTCG-3′; |
| GAPDH-forward | 5′-GAGGCAGGGATGATGTTCTG-3′; |
| GAPDH-reverse | 5′-CAGCCTCAAGATCATCAGCA-3′. |

XBP1: X-box binding protein 1; BLIMP-1: B lymphocyte-induced maturation protein-1; STAT-3: Signal transducer and activator of transcription 3; STAT-6: Signal transducer and activator of transcription 6; BLC-6: B-cell lymphoma 6; GAPDH: Glyceraldehyde 3-phosphate dehydrogenase
