# Supplementary material for: Inhibition of pathologic immunoglobulin E in food allergy by EBF-2 and active compound berberine associated with immunometabolism regulation
Source: Front Immunol. 2023 Feb 7;14:1081121. doi: 10.3389/fimmu.2023.1081121 (PMC9941740; doi:10.3389/fimmu.2023.1081121)
Supplement: Supplementary file 4 [file Table_3.docx]

**Supplemental Table 3. HPLC major peak intensities comparison**.

|  | **FAHF-2** | | | **EBF-2** | | |
| --- | --- | --- | --- | --- | --- | --- |
| Name | Retention time | Peak area (Mean ± SED) | % Peak area (Mean ± SED) | Retention Time | Peak area (Mean ± SED) | % Area (Mean ± SED) |
| Peak1 | 7.73 | 5225765.33±1996462.44 | 7.18±3.88 | ND | ND | ND |
| Peak2 | 8.214 | 557484.33± 328177.32 | 0.78±0.59 | 7.782 | 1075495.67±516947.4 | 0.95±0.46 |
| Peak3 | 10.081 | 1816480.33±813684.44 | 2.34±0.98 | 10.942 | 924859±482133.05 | 0.8±0.41 |
| Peak4 | 15.084 | 394802±104825.75 | 0.47±0.08 | ND | ND | ND |
| Peak5 | 18.65 | 698406.67±410908.8 | 0.91±0.52 | 18.84 | 1180235±350322.03 | 1.01±0.19 |
| Peak6 | 19.506 | 461071.33±116832.94 | 0.62±0.27 | 19.195 | 350115±27475.34 | 0.32±0 |
| Peak7 | 22.172 | 751192±255338.54 | 0.95±0.18 | 23.227 | 771828.67±181028.81 | 0.69±0.24 |
| Peak8 | 25.566 | 888258±343523.62 | 1.12±0.26 | 25.935 | 2102346.67±1049393.2 | 1.78±0.82 |
| Peak9 | 33.48 | 448802±102808.32 | 0.58±0.06 | 32.479 | 818952.33±474156.15 | 0.69±0.39 |
| Peak10 | 35.547 | 736955.5±177730.58 | 0.87±0.13 | 33.668 | 559208.67±218504.27 | 0.5±0.25 |
| Peak11 | 36.721 | 6301026.5±300494.22 | 7.54±1.06 | 37.759 | 8305970.33±4849819.38 | 6.94±3.33 |
| Peak12 | 37.542 | 2339886.33±494730.62 | 3.01±0.33 | 38.308 | 3962237.67±1693716.87 | 3.35±1.22 |
| **Peak13** | **41.087** | **26173262±8201440.12** | **33.26±6.40** | **42.018** | **72941236±12299148.43*** | **62.83±3.53*** |
| Peak15 | 44.102 | ND | ND | 45.693 | 1668082±176178.53 | 1.44±0.07 |
| Peak16 |  | ND | ND | 47.006 | 630692.33±112457.37 | 0.56±0.16 |
| Peak17 | 48.052 | 590500±141362.79 | 0.77±0.17 | ND | ND | ND |
| Peak18 | 56.041 | 1014807±366366.74 | 1.36±0.65 | 56.157 | 665317±227512.91 | 0.59±0.26 |
| Peak20 | 58.898 | 452391±128869.51 | 0.6±0.23 | ND | ND | ND |
| Peak21 | 60.213 | 749668.67±235037.8 | 1±0.43 | 60.337 | 436094±76460.87 | 0.39±0.13 |
| Peak22 | 60.877 | 636032.67±200050.24 | 0.85±0.36 | 60.998 | 354437±29894.58 | 0.31±0.06 |
| Peak23 | 61.903 | 1068061±331070.96 | 1.43±0.62 | 62.032 | 614916.33±156126.88 | 0.54±0.17 |
| Peak24 | 64.496 | 696997.5±208575.99 | 0.97±0.50 | ND | ND | ND |
| Peak25 | 66.787 | 1548004.33±686469.72 | 2.08±1.15 | 66.92 | 1186353.33±363281.31 | 1.06±0.44 |
| Peak27 | 67.333 | 765277.33±250539.6 | 1.02±0.45 | 67.475 | 400072±39177.96 | 0.36±0.09 |
| Peak29 | 73.444 | 1278177.67±228281.66 | 1.69±0.48 | 73.562 | 742591.67±104525.61 | 0.65±0.13 |
| Peak30 | 74.771 | 535491±130698.36 | 0.69±0.14 | ND | ND | ND |
| Peak31 | 75.578 | 1325359.67±121002.29 | 1.75±0.42 | 75.712 | 497548.67±50907.94 | 0.43±0.06 |

The retention time, peak intensity, and peak area ratio of detected peaks in FAHF-2 and E-B-FAHF-2 formulas were analyzed by HPLC. Peak 13 was identified as berberine, a major compound present in both FAHF-2 and E-B-FAHF-2. Peak area and present of area (single peak area/ total area of the formula) were shown as means ± SEM, n=3. Both peak area and % of peak area of peak 13 were significantly higher in EBF-2 that FAHF-2. *p<0.05). N/D: not detected.
